# Epigenetic modulation of type-1 diabetes via a dual effect on pancreatic macrophages and β cells

**DOI:** 10.7554/eLife.04631

**Published:** 2014-11-19

**Authors:** Wenxian Fu, Julia Farache, Susan M Clardy, Kimie Hattori, Palwinder Mander, Kevin Lee, Inmaculada Rioja, Ralph Weissleder, Rab K Prinjha, Christophe Benoist, Diane Mathis

**Affiliations:** 1 Division of Immunology, Department of Microbiology and Immunobiology, Harvard Medical School, Boston, United States; 2 Center for Systems Biology, Massachusetts General Hospital, Harvard Medical School, Boston, United States; 3 Epinova DPU, Immuno-Inflammation Therapy Area, Medicines Research Centre, GlaxoSmithKline, Stevenage, United Kingdom; 4 Evergrande Center for Immunologic Diseases, Harvard Medical School and Brigham and Women's Hospital, Boston, United States; Osaka University, Japan

**Keywords:** autoimmune diabetes, bromodomain inhibitor, NF-KB, mouse

## Abstract

Epigenetic modifiers are an emerging class of anti-tumor drugs, potent in multiple cancer contexts. Their effect on spontaneously developing autoimmune diseases has been little explored. We report that a short treatment with I-BET151, a small-molecule inhibitor of a family of bromodomain-containing transcriptional regulators, irreversibly suppressed development of type-1 diabetes in NOD mice. The inhibitor could prevent or clear insulitis, but had minimal influence on the transcriptomes of infiltrating and circulating T cells. Rather, it induced pancreatic macrophages to adopt an anti-inflammatory phenotype, impacting the NF-κB pathway in particular. I-BET151 also elicited regeneration of islet β-cells, inducing proliferation and expression of genes encoding transcription factors key to β-cell differentiation/function. The effect on β cells did not require T cell infiltration of the islets. Thus, treatment with I-BET151 achieves a ‘combination therapy’ currently advocated by many diabetes investigators, operating by a novel mechanism that coincidentally dampens islet inflammation and enhances β-cell regeneration.

**DOI:** http://dx.doi.org/10.7554/eLife.04631.001

## eLife digest

The DNA inside a cell is often tightly wrapped around proteins to form a compact structure called chromatin. Chemical groups added to the chromatin can encourage nearby genes to either be switched on or off; and several enzymes and other proteins help to read, add, or remove these marks from the chromatin. If these chromatin modifications (or the related enzymes and proteins) are disturbed it can lead to diseases like cancer. It has also been suggested that similar changes may influence autoimmune diseases, in which the immune system attacks the body's own tissues.

Drugs that target the proteins that read, add, or remove these chromatin modifications are currently being developed to treat cancer. For example, drugs that inhibit one family of these proteins called BET have helped to treat tumors in mice that have cancers of the blood or lymph nodes. However, because these drugs target pathways involved in the immune system they may also be useful for treating autoimmune diseases.

Now Fu et al. have tested whether a BET inhibitor might be a useful treatment for type-1 diabetes. In patients with type-1 diabetes, the cells in the pancreas that produce the insulin hormone are killed off by the immune system. Without adequate levels of insulin, individuals with type-1 diabetes may experience dangerous highs and lows in their blood sugar levels and must take insulin and sometimes other medications.

Using mice that spontaneously develop type-1 diabetes when still relatively young, Fu et al. tested what would happen if the mice received a BET inhibitor for just 2 weeks early on in life. Treated mice were protected from developing type-1 diabetes for the rest of their lives. Specifically, the treatment protected the insulin-producing cells and allowed them to continue producing insulin. The drug reduced inflammation in the pancreas and increased the expression of genes that promote the regeneration of insulin-producing cells.

Diabetes researchers have been searching for drug combinations that protect the insulin-producing cells and boost their regeneration. As such, Fu et al. suggest that these findings justify further studies to see if BET inhibitors may help to treat or prevent type-1 diabetes in humans.

**DOI:** http://dx.doi.org/10.7554/eLife.04631.002

## Introduction

Acetylation of lysine residues on histones and non-histone proteins is an important epigenetic modification of chromatin (Kouzarides, 2000). Multiple ‘writers’, ‘erasers’, and ‘readers’ of this modification have been identified: histone acetyltransferases (HATs) that introduce acetyl groups, histone deacetylases (HDACs) that remove them, and bromodomain (BRD)-containing proteins that specifically recognize them. Chromatin acetylation impacts multiple fundamental cellular processes, and its dysregulation has been linked to a variety of disease states, notably various cancers (Dawson and Kouzarides, 2012). Not surprisingly, then, drugs that modulate the activities of HATs or HDACs or, most recently, that block acetyl-lysine:BRD interactions are under active development in the oncology field.

BRDs, conserved from yeast to humans, are domains of approximately 110 amino-acids that recognize acetylation marks on histones (primarily H3 and H4) and certain non-histone proteins (e.g., the transcription factor, NF-κB), and serve as scaffolds for the assembly of multi-protein complexes that regulate transcription (Dawson et al., 2011; Prinjha et al., 2012). The BET subfamily of BRD-containing proteins (BRDs 2, 3, 4 and T) is distinguished as having tandem **b**romodomains followed by an ‘**e**xtra-**t**erminal’ domain. One of its members, Brd4, is critical for both ‘bookmarking’ transcribed loci post-mitotically (Zhao et al., 2011) and surmounting RNA polymerase pausing downstream of transcription initiation (Jang et al., 2005; Hargreaves et al., 2009; Anand et al., 2013; Patel et al., 2013).

Recently, small-molecule inhibitors of BET proteins, for example, JQ1 and I-BET, were found to be effective inhibitors of multiple types of mouse tumors, including a NUT midline carcinoma, leukemias, lymphomas and multiple myeloma (Filippakopoulos et al., 2010; Dawson et al., 2011; Delmore et al., 2011; Zuber et al., 2011). A major, but not the unique, focus of inhibition was the Myc pathway (Delmore et al., 2011; Mertz et al., 2011; Zuber et al., 2011; Lockwood et al., 2012). In addition, BET-protein inhibitors could prevent or reverse endotoxic shock induced by systemic injection of bacterial lipopolysaccharide (LPS) (Nicodeme et al., 2010; Seal et al., 2012; Belkina et al., 2013). The primary cellular focus of action was macrophages, and genes induced by the transcription factor NF-κB were key molecular targets (Nicodeme et al., 2010; Belkina et al., 2013).

Given several recent successes at transposing drugs developed for cancer therapy to the context of autoimmunity, it was logical to explore the effect of BET-protein inhibitors on autoimmune disease. We wondered how they might impact type-1 diabetes (T1D), hallmarked by specific destruction of the insulin-producing β cells of the pancreatic islets (Bluestone et al., 2010). NOD mice, the ‘gold standard’ T1D model (Anderson and Bluestone, 2005), spontaneously and universally develop insulitis at 4–6 weeks of age, while overt diabetes manifests in a subset of individuals beginning from 12–15 weeks, depending on the particular colony. NOD diabetes is primarily a T-cell-mediated disease, but other immune cells—such as B cells, natural killer cells, macrophages (MFs) and dendritic cells (DCs)—also play significant roles. We demonstrate that a punctual, 2-week, treatment of early- or late-stage prediabetic NOD mice with I-BET151 affords long-term protection from diabetes. Mechanistic dissection of this effect revealed important drug influences on both MFs and β cells, in particular on the NF-κB pathway. On the basis of these findings, we argue that epigenetic modifiers are an exciting, emerging option for therapeutic intervention in autoimmune diabetes.

## Results

### I-BET151 protects NOD mice from development of diabetes

T1D progresses through identifiable phases, which are differentially sensitive to therapeutic intervention (Bluestone et al., 2010). Therefore, we treated NOD mice with the BET-protein inhibitor, I-BET151 (GSK1210151A [Dawson et al., 2011; Seal et al., 2012]) according to three different protocols: from 3–5 weeks of age (incipient insulitis), from 12–14 weeks of age (established insulitis), or for 2 weeks beginning within a day after diagnosis of hyperglycemia (diabetes). Blood-glucose levels of insulitic mice were monitored until 30 weeks of age, after which animals in our colony generally do not progress to diabetes.

**Figure 1.**
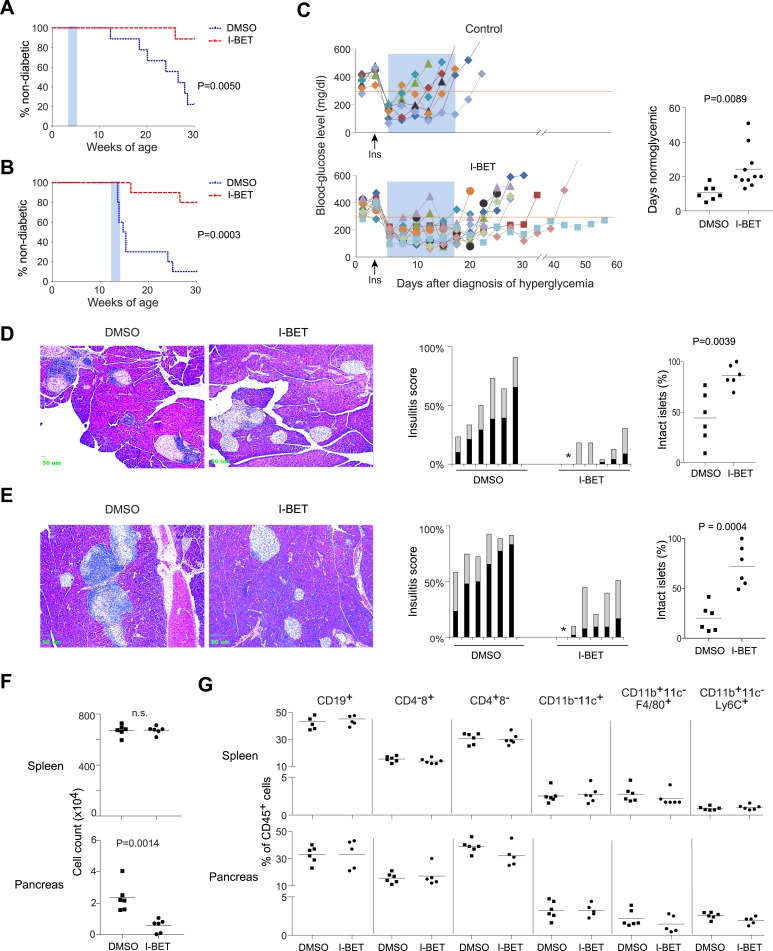
I-BET151 inhibits diabetes and insulitis in NOD mice. Female NOD mice were treated with I-BET151 in DMSO (10 mg/kg daily) or just DMSO from 3–5 weeks (**A** and **D**) or 12–14 weeks (**B** and **E**) of age. (**A** and **B**) Pre-diabetic mice. Hyperglycemia was monitored until 30 weeks of age. n = 10 per group. Blue shading = treatment window. (**C**) Recent-onset diabetic mice. Left: Individual blood-glucose curves. An insulin pellet was implanted subcutaneously within 1 day of diabetes diagnosis (arrow). 2 days later, I-BET151 (10 mg/kg) or DMSO was administered daily for 2 weeks (shaded blue). Right: duration of normoglycemia. n = 7 or 11. (**D** and **E**) Insulitis was visualized by H&E staining of paraffin sections. Left: representative histology. Middle: insulitis scores for individual mice. Grey = peri-insulitis; black = insulitis. The asterisk indicates no insulitis in any of the sections examined. Right: summary of the proportions of intact islets for individual mice. (**F**) Left: Total CD45^+^ cells from the spleen (upper panel) or pancreatic islets (lower panel) from mice treated with I-BET151 or DMSO as per Figure 1B, and analyzed at 14 weeks. n = 6. (**G**) Summary data on the major immune-cell subsets as a fraction of CD45^+^ cells, from the spleen (upper panels) or pancreas (lower panels). n = 5 or 6. p values in **A** and **B** are from Gehan-Breslow-Wilcoxon tests and in **C**–**G** are from Student's *t* tests. **DOI:** http://dx.doi.org/10.7554/eLife.04631.003

I-BET151 prevented diabetes development, no matter whether the treated cohort had incipient (Figure 1A) or established (Figure 1B) insulitis. However, the long-term protection afforded by a 2-week treatment of pre-diabetic mice was only rarely observed with recent-onset diabetic animals. Just after diagnosis, individuals were given a subcutaneous insulin implant, which lowers blood-glucose levels to the normal range within 2 days, where they remain for only about 7 days in the absence of further insulin supplementation (Figure 1C, upper and right panels). Normoglycemia was significantly prolonged in mice treated for 2 weeks with I-BET151; but, upon drug removal, hyperglycemia rapidly ensued in most animals (Figure 1C, lower and right panels). The lack of disease reversal under these conditions suggests that β-cell destruction had proceeded to the point that dampening the autoinflammatory attack was not enough to stem hyperglycemia. However, there was prolonged protection from diabetes in a few cases, suggesting that it might prove worthwhile to explore additional treatment designs in future studies.

As a first step in dissecting the mechanisms of I-BET151 action, we examined its effect on insulitis. Analogous to the protocols employed above, NOD mice were treated with I-BET151 from 3–5 or 12–14 weeks of age, and their pancreas was excised for histology at 10 weeks (5 weeks being too early for quantification) or 14 weeks, respectively. Drug treatment prevented effective installation of insulitis in the young mice (Figure 1D) and reversed established insulitis in the older animals (Figure 1E).

Next, we performed flow cytometric analysis of the pancreatic infiltrate. In this and subsequent mechanistic studies, we focused mainly on the 12–14-week treatment protocol because, of the successful regimens, it better models what might eventually be applied to humans. Consistent with the histological results, fewer total leukocytes (CD45^+^ cells) were found in the pancreas, but not the spleen, of mice administered I-BET151 from 12–14 weeks of age, *vis a vis* vehicle-only controls (Figure 1F). The drop in pancreas-infiltrating cells in animals treated with the inhibitor was equally true of all populations examined (encompassing the major lymphoid and myeloid subsets) as their fractional representation within the bulk CD45^+^ compartment appeared to be unaltered in drug- vs vehicle-treated individuals (Figure 1G).

### BET protein inhibition has a minimal effect on T cells in NOD mice

**Figure 2.**
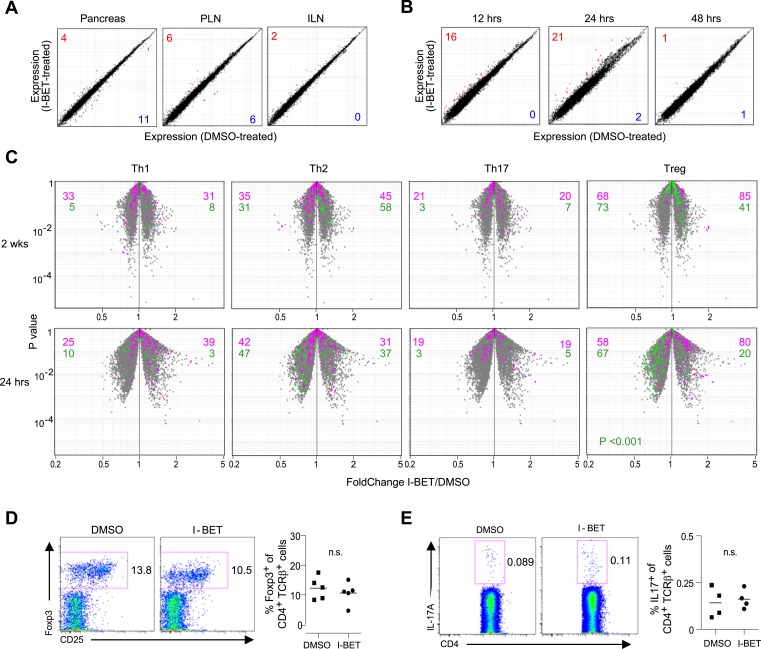
Little impact of BET-protein inhibition on CD4^+^ T cells in NOD mice. (**A**) Microarray-based transcriptional profiling of TCR^+^CD4^+^ cells sorted from pancreata, pancreatic lymph nodes (PLNs) and inguinal lymph nodes (ILNs). Comparison plot of I-BET151- and DMSO-treated mice as per Figure 1B and analyzed at 14 weeks of age. Red, transcripts increased >twofold by I-BET151; blue, transcripts >twofold decreased. (**B**) Analogous plots of TCR^+^CD4^+^ cells sorted from the pancreas of mice given a single I-BET151 (10 mg/kg) or DMSO injection, and analyzed 12, 24 or 48 hr later. (**C**) Th1, Th2, Th17 or Treg signatures (see ‘Materials and methods’) were superimposed on volcano plots comparing the transcriptomes of TCR^+^CD4^+^ cells from the pancreas of mice treated with I-BET151 or DMSO either as per Figure 1B and analyzed at 14 weeks of age (upper panels) or with a single injection and analyzed 24 hr later (lower panels). Purple: over-represented signature transcripts; Green: under-represented signature transcripts. (**D** and **E**) Proportions of Treg (**D**) or Th17 (**E**) cells within the TCR^+^CD4^+^ population in the pancreas of I-BET151- or DMSO-treated mice. Left, representative cytofluorometric dot plots; right, summary data. n = 4–5. p values in panel **C** are from the Chi-squared test (the single significant value is shown; all others were not significant) and in **D**–**E** from Student's *t* tests. **DOI:** http://dx.doi.org/10.7554/eLife.04631.004

Given that NOD diabetes is heavily dependent on CD4^+^ T cells (Anderson and Bluestone, 2005), and that a few recent reports have highlighted an influence of BET-protein inhibitors on the differentiation of T helper (Th) subsets in induced models of autoimmunity (Bandukwala et al., 2012; Mele et al., 2013), we explored the effect of I-BET151 treatment on the transcriptome of CD4^+^ T cells isolated from relevant sites; that is, the infiltrated pancreas, draining pancreatic lymph nodes (PLNs), and control inguinal lymph nodes (ILNs). Microarray analysis of gene expression revealed surprisingly little impact of the 2-week treatment protocol on any of these populations, similar to what was observed when comparing randomly shuffled datasets (Figure 2A). It is possible that the above protocol missed important effects on T cells because those remaining after prolonged drug treatment were skewed for ‘survivors’. Therefore, we also examined the transcriptomes of pancreas-infiltrating CD4^+^ T cells at just 12, 24 or 48 hr after a single administration of I-BET151. Again, minimal, background-level, differences were observed in the gene-expression profiles of drug- and vehicle-treated mice (Figure 2B).

Signature analysis, wherein we superimposed existing Th1, Th2, Th17 or Treg gene-expression signatures on p-value vs fold-change (FC) volcano plots, failed to reveal statistically significant skewing within the transcriptomes of pancreatic CD4^+^ T cells from mice administered I-BET151 vs vehicle using either the 2-week or short-term protocols, with the possible exception of a slightly weaker Treg down-signature in I-BET151-treated animals (Figure 2C). Yet, we found no differences in the fraction of Tregs in the pancreatic CD4^+^ T cell compartment, their Foxp3 expression levels or their display of CD25 (Figure 2D). Nor, in contrast to a recent report on antigen + adjuvant induced autoimmune diseases (Mele et al., 2013), could we demonstrate differences in the Th17 population—either the fraction of CD4^+^ cells expressing IL-17A or its expression level (Figure 2E). It remains possible, however, that parameters we did not assay (e.g., splicing, microRNAs) might have been different.

### I-BET151 induces a regulatory phenotype in the pancreatic macrophage population

**Figure 3.**
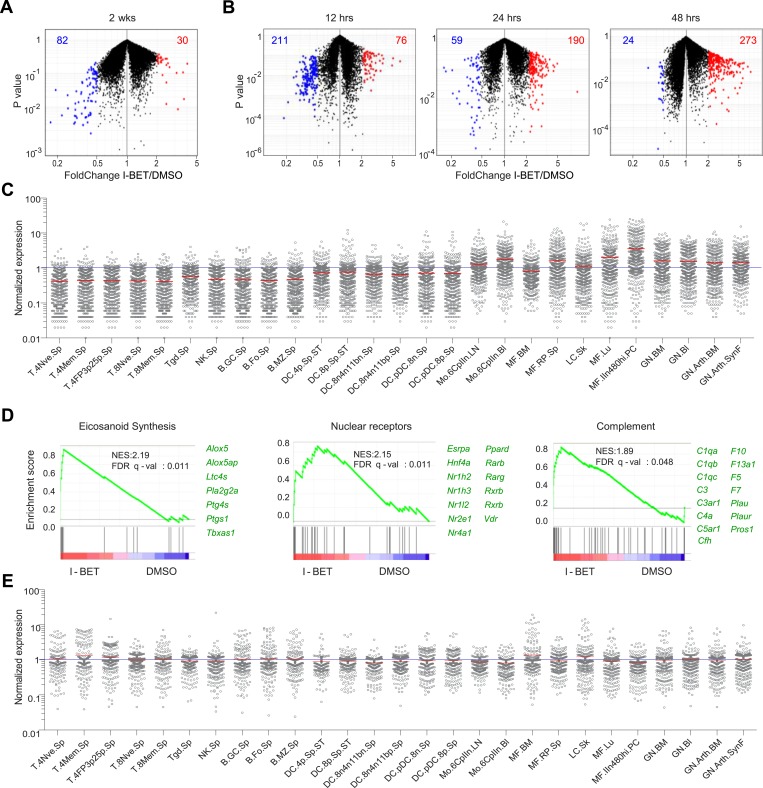
I-BET151 treatment promotes an MF-like, anti-inflammatory transcriptional program in pancreatic CD45^+^ cells. (**A** and **B**) A volcano plot comparing the transcriptomes of pancreatic CD45^+^ cells from mice treated with I-BET151 or DMSO as per Figure 1B. Red: transcripts increased >twofold; blue: transcripts decreased >twofold; numbers of modulated transcripts are indicated in the corresponding color. (**B**) Analogous plots for mice given a single injection of I-BET151 (10 mg/kg) or DMSO only, and analyzed 12, 24 or 48 hr later. (**C**) Cell-type distribution of the totality of transcripts whose expression was increased >twofold in panels **A** and **B** (red). Expression data for and definition of the various cell-types came from ImmGen (www.Immgen.org). Langerhans cells of the skin (LC.SK) have been re-positioned as per recent data (Gautier et al., 2012). Expression values were row-normalized. (**D**) GSEA of the totality of transcripts increased in pancreatic CD45^+^ cells of mice treated with I-BET151 (red dots in panels **A** and **B**). NES, normalized enrichment score. Representative genes showing increased expression on the right. (**E**) A plot analogous to that in panel **C** for the totality of transcripts >twofold under-represented in drug-treated mice (blue dots in panels **A** and **B**). **DOI:** http://dx.doi.org/10.7554/eLife.04631.005

To obtain a broader, unbiased view of the inhibitor's effect on the NOD pancreatic infiltrate, we undertook a transcriptome analysis of the bulk CD45^+^ cell population. The 2-week and short-term treatment protocols both resulted in sets of over- and under-represented transcripts (Figure 3A,B and Supplementary files 1, 2). Interestingly, the set of decreased transcripts was evident sooner than the set of increased transcripts—the former troughing already at 12 hr, the latter still rising at 48 hr (Figure 3B). Taking advantage of data-sets from the Immunological Genome Project (ImmGen; www.immgen.org), which has profiled gene expression for over 200 immunocyte populations, we found the over-represented transcripts to be indicative of myeloid-lineage cells, in particular tissue-resident MFs (Figure 3C). While it is possible that the changes in transcript levels reflect alterations in the relative representation of myeloid cell populations, we think rather that they resulted at least partially from gene induction or repression because they were so rapid and because only a fraction of the transcript set characteristic of any particular cell-type showed an altered level of expression. Gene-set enrichment analysis (GSEA) also highlighted transcriptional programs characteristic of MFs, the highest enrichment values being obtained for the eicosanoid, relevant nuclear receptor and complement pathways (Figure 3D). All three of these pathways have been demonstrated to play a role in the resolution of inflammation, through multiple mechanisms, including the production of anti-inflammatory mediators and suppression of T cell responses (Bensinger and Tontonoz, 2008; Ricklin et al., 2010; Serhan, 2011; Lone and Tasken, 2013). Parallel analyses on the set of pancreatic CD45^+^ cell transcripts under-represented in inhibitor-treated mice showed no striking enrichment across the ImmGen data-sets (Figure 3E). And GSEA did not reveal any particular pathways to be significantly enriched.

**Figure 4.**
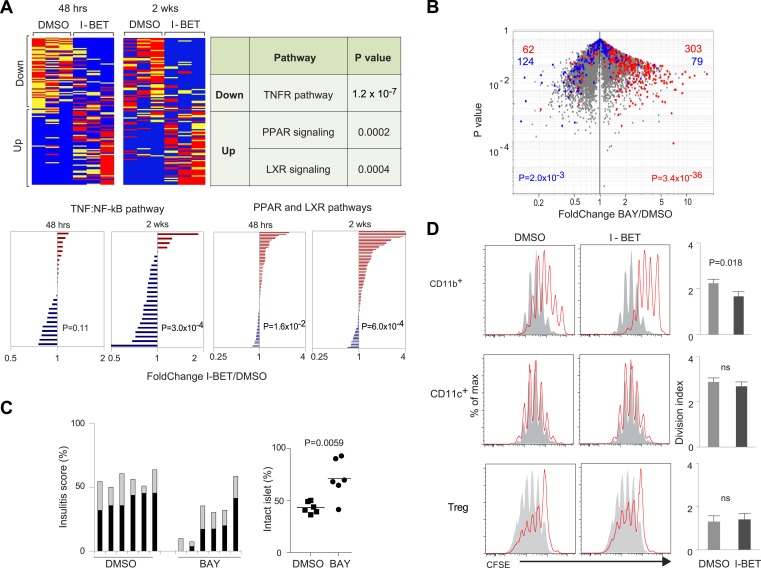
The NF-κB signaling pathway is a major focus of I-BET151's influence on NOD leukocytes. (**A**) Upper panels: The inhibitor's effect on NF-κB-regulated genes—defined as per http://www.bu.edu/nf-kb/gene-resources/target-genes. Left, relevant transcripts from pancreatic CD45^+^ cells of NOD mice treated long- or short-term with I-BET151 or DMSO. Red: over-represented; blue: under-represented. 2 wk: long-term, treatment as per Figure 1B; 48 hr: short-term, treatment with a single 10 mg/kg dose and analyzed 48 hr later. Right, signaling pathways represented by the enriched or impoverished transcripts in the data to the left, via Ingenuity pathway analysis (www.ingenuity.com). Lower panels: Gene sets corresponding to the TNFα-induced canonical NF-κB pathway (Schaefer et al., 2009) or the PPAR and LXR pathways (http://www.genome.jp/kegg/pathway/hsa/hsa03320.html) were retrieved from the Broad Institute's Molecular Signatures Database (http://www.broadinstitute.org/gsea/msigdb), and their expression levels in CD45^+^ cells from pancreas of I-BET151- or vehicle-treated mice plotted. (**B**) 12-week-old NOD mice were injected once ip with BAY 11–7082 (10 mg/kg), sacrificed 24 hr later, and CD45^+^ cells from the pancreas isolated and transcriptionally profiled. A volcano plot comparing treatment with BAY 11–7082 and DMSO, with genes >twofold increased (in red) or decreased (in blue) by I-BET151 treatment (pooled from all time-points of Figure 3A,B) superimposed. (**C**) Effect of a single dose of 10 mg/kg BAY 11–7082 on insulitis in 12-week-old NOD mice, analyzed 24 hr after injection. Left: insulitis scores. Right: summary data for the fraction of islets with no infiltrate. Grey, peri-insulitis; Black, insulitis. (**D**) Suppression of in vitro T cell proliferation by cell populations isolated from the pancreas of I-BET151- or DMSO- treated mice (as per Figure 1B). The CD11b^+^CD11c^−^ (top), CD11b^−^CD11c^+^ (middle) and TCRβ^+^CD4^+^CD25^+^ (bottom) fractions of CD45^+^ cells were sorted. To the left are representative plots of CFSE dilution; to the right are summary data quantifying division indices (see ‘Materials and methods’ for details). p values in **A** and **B** are from the chi-squared test, and in **C** and **D** are from the Student's *t* test. **DOI:** http://dx.doi.org/10.7554/eLife.04631.006

We then focused specifically on the impact of I-BET151 on the NF-κB pathway in pancreatic leukocytes, with the following considerations in mind: (1) NF-κB is a critical player in many types of inflammation, exerting both pro- and anti-inflammatory influences (Vallabhapurapu and Karin, 2009); (2) specifically, NF-κB has been implicated in T1D (Rink et al., 2012); (3) direct binding of a BET protein, Brd4, to the RelA subunit of NF-κB has been documented (Huang et al., 2009; Zhang et al., 2012; Zou et al., 2014); and (4) BET inhibitors are known to influence the NF-κB-induced transcriptional program in MFs (Nicodeme et al., 2010). Interestingly, both 2-week and short-term treatment of NOD mice with I-BET151 had a dichotomous effect on the NF-κB target genes expressed by pancreatic leukocytes (Figure 4A, upper left panels, and Supplementary file 3; target genes extracted from [Gilmore, 2006]). Pathway analysis using Ingenuity showed that, amongst these NF-κB targets, pro-inflammatory genes, particularly those encoding proteins in the tumor necrosis factor (TNF)α-induced canonical NF-κB activation pathway, were under-represented; while anti-inflammatory loci, notably those specifying molecules implicated in signaling by nuclear receptor family members, that is, the peroxisome proliferator-activated receptor (PPAR) and liver-X-receptor (LXR) families, were over-represented subsequent to I-BET151 treatment (Figure 4A, upper right). These findings were confirmed by examining expression of pathway signature genes derived from the Molecular Signatures Database (www.broadinstitute.org/gsea/msigdb): again the TNF-induced NF-κB activation pathway was down-regulated and the two nuclear receptor pathways up-regulated (Figure 4A, lower panel). There were no such effects on CD45^+^ cells isolated from the spleen or lymph nodes of the same mice.

We evaluated to what extent an NF-κB inhibitor could mimic the effects of I-BET151. Given its greater toxicity, a single dose of BAY 11–7082 (which blocks IκBα phosphorylation) was administered to 12-week-old NOD mice, and pancreatic CD45^+^ cells were isolated for transcriptome analysis 24 hr later. When the sets of genes over- or under-represented in the CD45^+^ population of I-BET151-treated mice (drawn from Figure 3A,B) were superimposed on a volcano plot of transcripts from CD45^+^ cells of animals given BAY 11–7082 vs vehicle, there was an impressive correspondence, particularly for the enriched transcripts (Figure 4B). The match was not perfect, however, as some genes modulated by the BET-protein inhibitor were not affected by the NF-κB inhibitor and vice versa (Figure 4B). Perhaps not surprisingly, then, BAY 11–7082, even a single dose, could substantially clear NOD insulitis (Figure 4C). (Note that it was not possible to assess the effect of this drug on diabetes development because its toxicity precluded multiple administrations.)

We performed co-culture experiments to confirm the disease-dampening potential of pancreatic MFs from mice administrated BET inhibitor. As T cells are key orchestrators of NOD diabetes (Anderson and Bluestone, 2005), we examined the ability of various cell-types isolated from the pancreas of mice treated with I-BET151 or vehicle to impact an in vitro T cell proliferation assay, quantifying CFSE dilution of CD3/CD28-stimulated cells (Figure 4D). The CD11b^+^ population from the pancreas of vehicle-treated mice was capable of repressing T cell proliferation, but the corresponding population from mice administered I-BET151 exhibited significantly more potent suppressive activity. Co-cultured CD11c^+^ cells did not appear to affect T cell proliferation whether isolated from control- or drug-treated mice. Treg cells isolated from the two types of mice were equally able to inhibit T cell proliferation.

**Figure 5.**
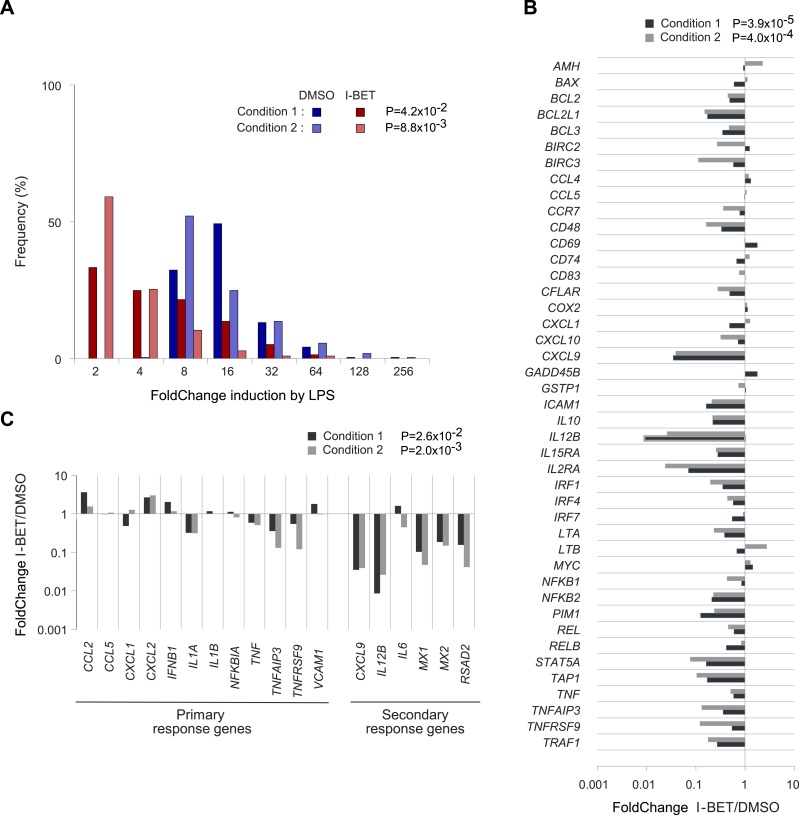
Effects of I-BET151 on human monocyte-derived MFs. Cultures of human MFs were differentiated from peripheral-blood-derived CD14^+^ cells, pre-cultured for 30 min with I-BET151 (red bars) or just DMSO (blue bars), and stimulated for another 4 hr after the addition of LPS (100 ng/ml) or vehicle only. Microarray data from two conditions, as indicated and detailed in ‘Materials and methods’. (**A**) Distribution of FCs of I-BET151/DMSO for LPS-induced genes (according to the data on human monocyte-derived MFs treated with LPS or just vehicle). (**B**) FCs of I-BET151/DMSO of the human orthologues of the set of murine NF-κB-regulated genes illustrated in Figure 4A. (**C**) Effects of I-BET151 on primary and secondary LPS-response genes (defined as per [Hargreaves et al., 2009; Ramirez-Carrozzi et al., 2009]). **DOI:** http://dx.doi.org/10.7554/eLife.04631.007

It was obviously of interest to see whether I-BET151 exerted similar effects on human MFs. CD14^+^ cells pooled from the peripheral blood of three donors were differentiated in culture; they were then incubated with I-BET151 or vehicle for 30 min, before stimulation with LPS for 4 hr; at which time, RNA was isolated for transcriptional profiling. A rank-order FC plot showed that the inhibitor greatly dampened the typical MF response to LPS (Figure 5A). Focusing on the NF-κB pathway: most (32/43) of the target genes inhibited in murine CD45^+^ cells were also suppressed in human MFs (Figure 5B). In particular, there was suppression of secondary, over primary, LPS-response genes (Figure 5C), as was previously reported for BET inhibitor treatment of mouse bone-marrow-derived MFs (Nicodeme et al., 2010).

### BET-protein inhibition promotes regeneration of NOD β cells

**Figure 6.**
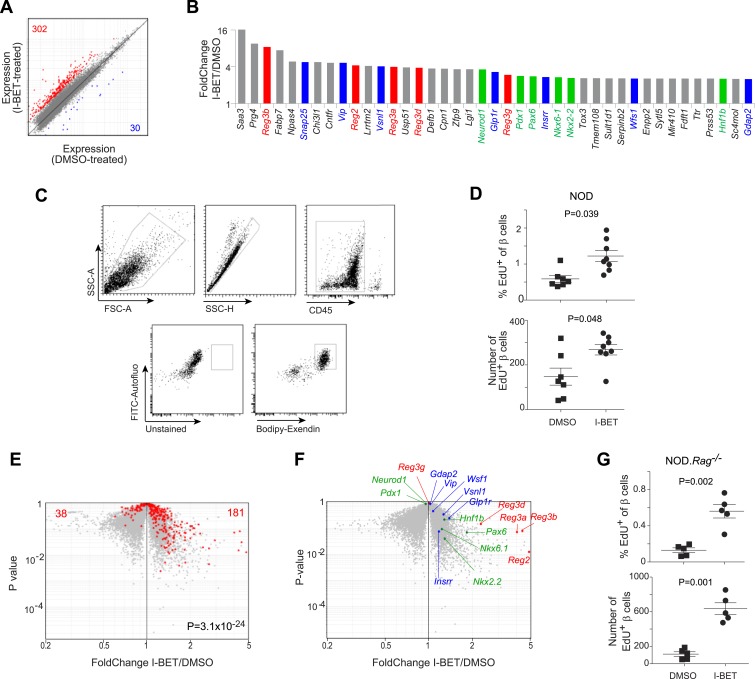
BET-protein inhibition promotes regeneration of islet β cells. (**A**) Pancreatic β cells were cytofluorometrically sorted from mice treated with I-BET151 or DMSO as per Figure 1B, and microarray-based transcriptional profiling performed. Red: transcripts increased >twofold by I-BET151; blue, transcripts >twofold decreased. (**B**) NOD β-cell transcripts increased by I-BET151 ranked by FC vis-à-vis DMSO treatment. Red, regenerating islet-derived (Reg) transcripts; green, transcripts encoding transcription factors important for β-cell differentiation and function; blue, transcripts encoding proteins that enhance insulin production. (**C**, **D**, **G**) Cytofluorometric quantification of EdU^+^ β cells from NOD (**D**) or NOD.Rag^−/−^ (**G**) mice treated with I-BET151 or DMSO as in Figure 1B and injected with EdU during the last 24 hr n = 5–8. Panel **C** shows the sorting strategy. (**E**) The set of red transcripts from panel **A** was superimposed on volcano plots comparing gene expression by β cells from NOD.Rag^−/−^ mice treated as in Figure 1B with I-BET151 vs DMSO. (**F**) Relevant I-BET151-induced transcripts highlighted in panel **B** are situated on the volcano plot of panel **E**. (**G**) p values: *<0.05; **<0.01; *<0.001—from the Student's *t* test for panels **D** and **G**, and from the chi-squared test for panel **E**. **DOI:** http://dx.doi.org/10.7554/eLife.04631.008

We questioned whether the disease-protective effect of I-BET151 also entailed an influence on the target of the autoimmune attack, pancreatic islet β cells. As an initial approach, we compared global gene-expression profiles of flow-cytometrically purified β cells from NOD mice treated from 12–14 weeks of age with I-BET151 or vehicle. A set of about 300 transcripts was over-represented >twofold in mice given the inhibitor; and about 10-fold fewer were under-represented >twofold (FDR < 5%) (Figure 6A and Supplementary file 4). The subset of genes whose expression was increased most by the inhibitor encoded multiple members of the regenerating islet-derived (Reg) protein family, originally identified for their involvement in pancreas regeneration (Figure 6B, in red) (Unno et al., 1992; Huszarik et al., 2010); several transcription factors (TFs) important for regeneration of β-cells (in green) (Pagliuca and Melton, 2013; Shih et al., 2013); and a number of proteins known to enhance insulin production (in blue) (Hirayama et al., 1999; Dai et al., 2006; Winzell and Ahren, 2007). Interestingly, the most highly induced subset of genes also included a number of loci usually associated with neural function, encoding, for example, survival factors (*Cntfr, Tox3*) or promoters of synaptic development or function (*Snap25, Lrrtm2, Lgi1*). The top signaling pathways to emerge via GSEA were the IGF-1, IGF/mTOR, PDGF and insulin-secretion pathways (although, due to the relatively small number of genes involved, statistical significance was not reached).

**Figure 6—figure supplement 1.**
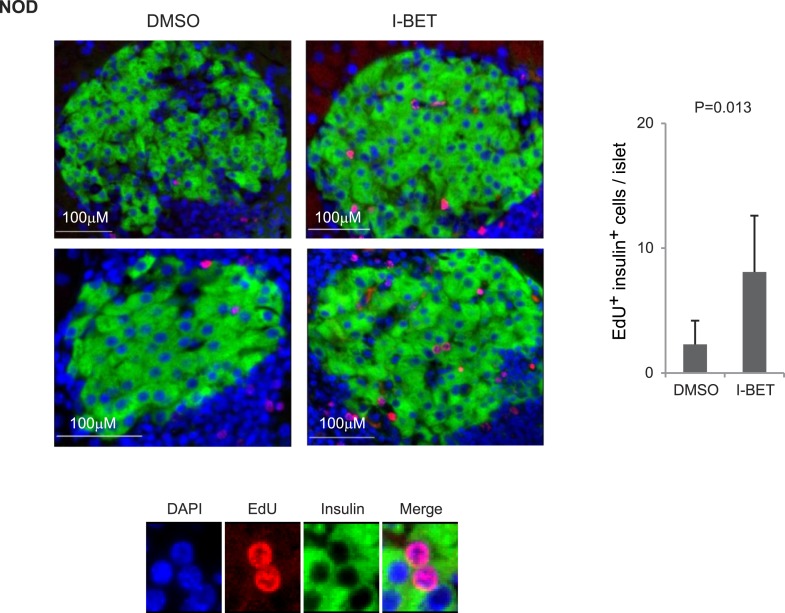
Histologic analysis of β-cell proliferation in response to I-BET151 in NOD mice. NOD mice were treated with I-BET151 or DMSO as in Figure 1B. EdU was administrated during the last 24 hr. Frozen sections of pancreas were stained for insulin and EdU. Left, representative islet images; two for each condition; right, summary quantification. Small images in color are legends for insulin, EdU and DAPI, respectively. Red, EdU; green, insulin; blue, DAPI. **DOI:** http://dx.doi.org/10.7554/eLife.04631.009

**Figure 6—figure supplement 2.**
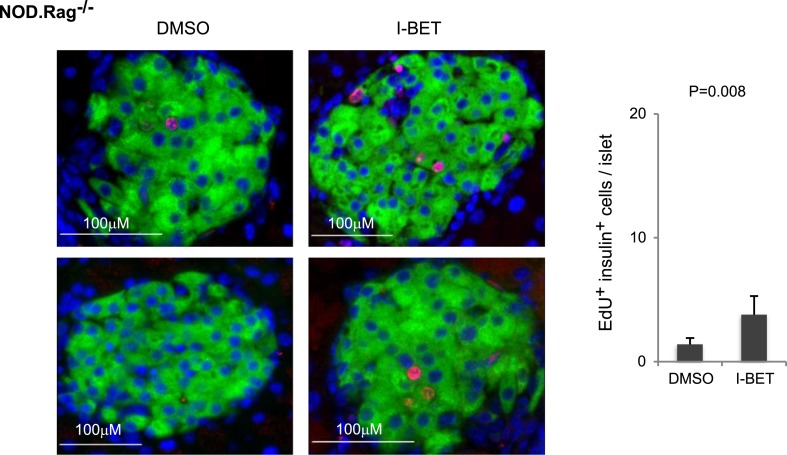
Histologic analysis of β-cell proliferation in response to I-BET151 in NOD.Rag^−/−^ mice. NOD.Rag^−/−^ mice were treated with I-BET151 or DMSO as in Figure 1B. EdU was administrated during the last 24 hr. Frozen sections of pancreas were stained for insulin and EdU. Left, representative islet images, two for each condition; right, summary quantification. **DOI:** http://dx.doi.org/10.7554/eLife.04631.010

The NF-κB pathway has been implicated in the death of β-cells associated with T1D, mainly the branches downstream of inflammatory cytokine and stress stimuli (Cardozo et al., 2001; Cnop et al., 2005; Eldor et al., 2006). Several NF-κB-dependent transcripts previously reported to be induced in β cells by the inflammatory cytokines IL-1β and IFN-γ (Cardozo et al., 2001) were suppressed by I-BET151, for example, *Icam1*, 1.45-fold; *Traf2*, 1.28-fold; *Fabp5*, 1.23-fold. Overall, then, I-BET151 appeared to have a positive impact on β-cell regeneration and/or function.

To determine whether BET-protein inhibition induced proliferation of β cells, we treated 12-week-old NOD mice for the usual 2 weeks with I-BET151 or vehicle, injected them with 5-ethyl-2-deoxyuridine EdU 24 hr before sacrifice, and quantified EdU incorporation by β cells, delineated by their high autofluorescence and strong staining with a fluorescently conjugated exendin peptide probe (Figure 6C). Both the number and fraction of EdU^+^ β cells were significantly increased in I-BET151-treated mice (Figure 6D). Analogous results were obtained using fluorescent microscopy to quantify EdU^+^ insulin-expressing β cells (Figure 6—figure supplement 1).

Since it has been reported that T cells in the immune infiltrate can induce β-cell replication (Sreenan et al., 1999; Sherry et al., 2006; Dirice et al., 2013), we repeated the preceding experiments on Rag1-deficient NOD mice in order to address the possibility of a secondary effect mediated through lymphocytes. Transcriptional analysis revealed that most of the β-cell transcripts over-represented >twofold by treatment of standard NOD mice with I-BET151 were also increased in inhibitor-injected NOD mice lacking T and B cells, and thereby devoid of insulitis (Figure 6E; and the transcripts highlighted in panel 6B are precisely positioned in panel 6F). In addition, there was a clear induction of β-cell proliferation in the absence of insulitis (Figure 6, panel G and Figure 6—figure supplement 2).

We attempted to further evaluate the cell autonomy of I-BET151's effects on β cells by purifying islets from 12-week-old NOD mice and culturing them for 24 hr in the presence of inhibitor or just vehicle. No consistent differences were observed under the two culture conditions. Not surprisingly, then, a similar experiment using commercially available human islets failed to show a repeatable difference when they were cultured in the presence and absence of I-BET151. Clearly, the islet isolation/culture conditions removed or destroyed a critical factor.

## Discussion

The studies presented here showed that treatment of NOD mice with the epigenetic modifier, I-BET151, for a mere 2 weeks prevented the development of NOD diabetes life-long. I-BET151 was able to inhibit impending insulitis as well as clear existing islet infiltration. The drug had a dual mechanism of action: it induced the pancreatic MF population to adopt an anti-inflammatory phenotype, primarily via the NF-κB pathway, and promoted β-cell proliferation (and perhaps differentiation). These findings raise a number of intriguing questions, three of which we address here.

First, why do the mechanisms uncovered in our study appear to be so different from those proposed in the only two previous reports on the effect of BET-protein inhibitors on autoimmune disease? Bandukwala et al. found that I-BET762 (a small-molecule inhibitor similar to I-BET151) altered the differentiation of Th subsets in vitro, perturbing the typical profiles of cytokine production, and reducing the neuropathology provoked by transfer of in-vitro-differentiated Th1, but not Th17, cells reactive to a peptide of myelin oligodendrocyte glycoprotein (Bandukwala et al., 2012). Unfortunately, with such transfer models, it is difficult to know how well the in vitro processes reflect in vivo events, and to distinguish subsidiary effects on cell survival and homing. Mele et al. reported that JQ1 primarily inhibited the differentiation of and cytokine production by Th17 cells, and strongly repressed collagen-induced arthritis and experimental allergic encephalomyelitis (Mele et al., 2013). However, with adjuvant-induced disease models such as these, it is difficult to discriminate influences of the drug on the unfolding of autoimmune pathology vs on whatever the adjuvant is doing. Thus, the very different dual mechanism we propose for I-BET151's impact on spontaneously developing T1D in NOD mice may reflect several factors, including (but not limited to): pathogenetic differences in induced vs spontaneous autoimmune disease models; our broader analyses of immune target cell populations; and true mechanistic differences between T1D and the other diseases. As concerns the latter, it has been argued that T1D is primarily a Th1-driven disease, with little, or even a negative regulatory, influence by Th17 cells (discussed in [Kriegel et al., 2011]).

Second, how does I-BET151's effect, focused on MFs and β cells, lead to life-long protection from T1D? MFs seem to play a schizophrenic role in the NOD disease. They were shown long ago to be an early participant in islet infiltration (Jansen et al., 1994), and to play a critical effector role in diabetes pathogenesis, attributed primarily to the production of inflammatory cytokines and other mediators, such as iNOS (Hutchings et al., 1990; Jun et al., 1999a, 1999b; Calderon et al., 2006). More recently, there has been a growing appreciation of their regulatory role in keeping diabetes in check. For example, the frequency of a small subset of pancreatic MFs expressing the complement receptor for immunoglobulin (a.k.a. CRIg) at 6–10 weeks of age determined whether or not NOD diabetes would develop months later (Fu et al., 2012b), and transfer of in-vitro-differentiated M2, but not M1, MFs protected NOD mice from disease development (Parsa et al., 2012).

One normally thinks of immunological tolerance as being the purview of T and B cells, but MFs seem to be playing the driving role in I-BET151's long-term immunologic impact on T1D. Chronic inflammation (as is the insulitis associated with T1D) typically entails three classes of participant: myeloid cells, in particular, tissue-resident MFs; lymphoid cells, including effector and regulatory T and B cells; and tissue-target cells, that is, islet β cells in the T1D context. The ‘flavor’ and severity of inflammation is determined by three-way interactions amongst these cellular players. One implication of this cross-talk is that a perturbation that targets primarily one of the three compartments has the potential to rebalance the dynamic process of inflammation, resetting homeostasis to a new level either beneficial or detrimental to the individual. BET-protein inhibition skewed the phenotype of pancreatic MFs towards an anti-inflammatory phenotype, whether this be at the population level through differential influx, efflux or death, or at the level of individual cells owing to changes in transcriptional programs. The ‘re-educated’ macrophages appeared to be more potent at inhibiting T cell proliferation. In addition, it is possible that MFs play some role in the I-BET151 influences on β-cell regeneration. The findings on Rag1-deficient mice ruled out the need for adaptive immune cells in the islet infiltrate for I-BET151's induction of β-cell proliferation, but MFs are not thought to be compromised in this strain. Relatedly, the lack of a consistent I-BET151 effect on cultured mouse and human islets might result from a dearth of MFs under our isolation and incubation conditions (e.g., [Li et al., 2009]). Several recent publications have highlighted a role for MFs, particularly M2 cells, in promoting regeneration of β cells in diverse experimental settings (Brissova et al., 2014; Xiao et al., 2014), a function foretold by the reduced β-cell mass in MF-deficient *Csf1*^*op/op*^ mice reported a decade ago (Banaei-Bouchareb et al., 2004).

Whether reflecting a cell-intrinsic or -extrinsic impact of the drug, several pro-regenerative pathways appear to be enhanced in β-cells from I-BET151-treated mice. Increased β-cell proliferation could result from up-regulation of the genes encoding Neurod1 (Kojima et al., 2003), GLP-1R (De Leon et al., 2003), or various of the Reg family members (Unno et al., 2002; Liu et al., 2008), the latter perhaps a consequence of higher IL-22R expression (Hill et al., 2013) (see Figure 6B and Supplementary file 4). Protection of β-cells from apoptosis is likely to be an important outcome of inhibiting the NF-κB pathway (Takahashi et al., 2010), but could also issue from enhanced expression of other known pro-survival factors, such as Cntfr (Rezende et al., 2007) and Tox3 (Dittmer et al., 2011) (see Figures 4 and 6B). Lastly, β-cell differentiation and function should be fostered by up-regulation of genes encoding transcription factors such as Neurod1, Pdx1, Pax6, Nkx6-1 and Nkx2-2. The significant delay in re-onset of diabetes in I-BET151-treated diabetic mice suggests functionally relevant improvement in β-cell function. In brief, the striking effect of I-BET151 on T1D development in NOD mice seems to reflect the fortunate concurrence of a complex, though inter-related, set of diabetes-protective processes.

Lastly, why does a drug that inhibits BET proteins, which include general transcription factors such as Brd4, have such circumscribed effects? A 2-week I-BET151 treatment might be expected to provoke numerous side-effects, but this regimen seemed in general to be well tolerated in our studies. This conundrum has been raised in several contexts of BET-inhibitor treatment, and was recently discussed at length (Shi and Vakoc, 2014). The explanation probably relates to two features of BET-protein, in particular Brd4, biology. First: Brd4 is an important element of so-called ‘super-enhancers’, defined as unusually long transcriptional enhancers that host an exceptionally high density of TFs—both cell-type-specific and general factors, including RNA polymerase-II, Mediator, p300 and Brd4 (Hnisz et al., 2013). They are thought to serve as chromatin depots, collecting TFs and coordinating their delivery to transcriptional start-sites via intra-chromosome looping or inter-chromosome interactions. Super-enhancers are preferentially associated with loci that define and control the biology of particular cell-types, notably developmentally regulated and inducible genes; intriguingly, disease-associated, including T1D-associated, nucleotide polymorphisms are especially enriched in the super-enhancers of disease-relevant cell-types (Hnisz et al., 2013; Parker et al., 2013). Genes associated with super-enhancers show unusually high sensitivity to BET-protein inhibitors (Chapuy et al., 2013; Loven et al., 2013; Whyte et al., 2013). Second: although the bromodomain of Brd4 binds to acetyl-lysine residues on histone-4, and I-BET151 was modeled to inhibit this interaction, it is now known to bind to a few non-histone chromosomal proteins as well, notably NF-κB, a liaison also blocked by BET-protein inhibitors (Huang et al., 2009; Zhang et al., 2012; Zou et al., 2014). Abrogating specific interactions such as these, differing according to the cellular context, might be the dominant impact of BET inhibitors, a scenario that would be consistent with the similar effects we observed with I-BET151 and BAY 11–7082 treatment. Either or both of these explanations could account for the circumscribed effect of I-BET151 on NOD diabetes. Additionally, specificity might be imparted by different BET-family members or isoforms—notably both Brd2 and Brd4 are players in MF inflammatory responses (Belkina et al., 2013). According to either of these explanations, higher doses might unleash a broader array of effects.

Viewed in the context of recent reports, our data point to NF-κB as a direct target of I-BET151. Traditionally, Brd4's impact on transcription has been thought to reflect its binding to histone acetyl-lysine residues, as a so-called ‘histone reader’ (Muller et al., 2011; Prinjha et al., 2012). Analogously, the influence of I-BET151 (and like drugs) on Brd4 function has generally been attributed to Brd4 interactions with acetylated histones (Muller et al., 2011; Prinjha et al., 2012). However, it is now clear that I-BET 151 directly targets Brd4's association with non-histone proteins as well. The best-studied example is NF-κB (Chen et al., 2005; Nowak et al., 2008; Huang et al., 2009; Zou et al., 2014). The two bromodomains of Brd4 cooperatively recognize RelA acetylated at the K310 position, and this interaction is blocked by drugs like I-BET151 and JQ1.

Of late, there has been substantial interest in treating individuals with, or at risk of, T1D with combination therapies. It would seem logical to design a combinatorial approach that targets two or more of the major players in disease—perhaps optimally, addressing elements of the innate immune system, adaptive immune system and islet target tissue. We have demonstrated that a single drug, the BRD blocker I-BET151, has a potent effect on T1D in pre-diabetic NOD mice by coincidentally influencing MFs and β cells. Recently, another drug from the cancer world, the HDAC inhibitor, vorinostat, was reported to inhibit T1D, by a seemingly different mechanism impacting a multiplicity of cell-types (Christensen et al., 2014). Thus, epigenetic modulators would seem to be exciting candidates to explore in human T1D patients.

## Materials and methods

### Mice and disease evaluation

NOD/Lt mice were bred under specific-pathogen–free conditions in our animal facility at the New Research Building of Harvard Medical School, cared for in accordance with the ethical guidelines of the Institutional Animal Care and Use Committee. Relevant studies were also conducted in accordance with GSK's Policy on the Care, Welfare and Treatment of Laboratory Animals. NOD.Cg-Rag1<tm1mom> mice were maintained in our lab's colony at Jackson Laboratory.

For the evaluation of diabetes, mice were monitored until 30 weeks of age by measuring urine- and blood-glucose levels, as described (Katz et al., 1993). Individuals with two consecutive measurements of a serum-glucose concentration above 300 mg/dl were considered diabetic. For the recent-onset cohorts: on the same day as diabetes diagnosis, individuals received a single subcutaneously implanted insulin pellet (LinShin, Toronto, Canada), which lowers the blood-glucose level to the normal range within 2 days.

For insulitis assessment, mice were euthanized, and their pancreas removed and fixed in 10% neutral-buffered formalin (Sigma–Aldrich, St. Louis, MO). Paraffin-embedded sections were cut into 6 μm sections with 150 μm between steps, and were stained with hematoxylin and eosin (H&E). Insulitis was scored as described (Katz et al., 1993).

### Small-molecule inhibitors

The BET-protein BRD inhibitor (I-BET151, GSK1210151A) was produced and handled as described (Dawson et al., 2011). For long-term in vivo treatment, mice were ip-injected with I-BET151 dissolved in DMSO at a dose of 10 mg/kg daily for 2 weeks beginning at the designated age.

The NF-κB inhibitor (BAY 11–7082) was purchased from Sigma and dissolved in DMSO as a stock solution of 10 mg/ml. For in vivo treatment, BAY was injected ip at a dose of 10 mg/kg 24 hr before sacrificing mice for analysis.

### Flow cytometry

For immunocytes, all staining began by incubating with a mAb directed against FcγR (2.4G2; BD Pharmingen, San Diego, CA). mAbs against the following antigens were used: CD45 (30-F11), CD4 (RM4-5), CD8 (53-6.7), CD19 (6D5), CD11b (M1/70), CD11c (G418), Ly6C (AL-21), F4/80 (BM8) and IL-17A (TC11-18H10.1), all from BioLegend (San Diego, CA); and FoxP3 (FJK-16s, eBioscience, San Diego, CA).

For β cells, we followed the method described in (Li et al., 2009) with modifications. Briefly, the pancreas was perfused via the common bile duct with 5 ml of a solution of collagenase P (1 mg/ml, Roche); and was then digested at 37°C for 15 min, followed by several steps of centrifugation and washing. On average, 80 islets were purified by hand-picking, and were then cultured in complete RPMI1640 medium with the fluorescent exendin-4 probe (100 nM). After 1 hr, islets were disrupted by treatment with trypsin-EDTA solution in a 37°C waterbath for 10 min. The single-cell suspensions containing β cells were analyzed by flow cytometry.

The exendin-4 probe, EP12-BTMR-X, was synthesized as previously reported (compound 17 in Table 1 of Clardy et al., 2014). Briefly, an azide-functionalized BODIPY TMR-X was conjugated to exendin-4 (HGEGTFTSDLSPraQMEEEAVRLFIEWLKNGGPSSGAPPPS) using microwave-assisted copper-catalyzed azide-alkyne Huisgen cycloaddition. The conjugate was purified by HPLC and characterized by MALDI-TOF mass spectrometry and an I^125^ binding assay.

To quantify proliferation in vivo, we ip-injected mice with 1 mg EdU 24 hr before sacrifice, and single-cell suspensions were prepared, fixed and permeabilized using the eBioscience Fixation/Permeabilization set (Cat# 00-5123, 00-5223). They were then stained using the EdU staining kit (Click-iT EdU Flow Cytometry Assay Kits; Invitrogen, Carlsbad, CA), following the users' manual.

### Microarray analysis

Pancreata were collected and digested for 30 min at 37°C with collagenase IV (1 mg/ml) and DNase I (10 U/ml). Single-cell suspensions were prepared and stained, and CD4^+^ or CD45^+^ cells were sorted into 500 ml TRIzol (Invitrogen) for RNA isolation. RNA was amplified in two rounds with MessageAmp aRNA (Ambion) and labeled with biotin (BioArray High Yield RNA Transcription Labeling; Enzo, Farmingdale, NY), and the resulting cRNA was hybridized to MoGene 1.0 ST (mouse) or huGene 1.0 ST (human) arrays (Affymetrix, Santa Clara, CA). Raw data were normalized by the robust multi-array average (RMA) algorithm implemented in the Expression File Creator module of the GenePattern genomic analysis platform (Reich et al., 2006). For pathway analysis, GSEA was used, as described (Subramanian et al., 2005). Gene-expression signatures of Th1, Th2 and Th17 cells derived from (Wu et al., 2010) and (Vehik and Dabelea, 2011); and signatures for Treg cells from (Fu et al., 2012a).

For microarray analysis of islet β cells, NOD or NOD.Rag^−/−^ mice were sacrificed, and collegenase P solution (0.5 mg/m) administered intrapancreatically via the bile duct. Pancreata were than digested for 20 min at 37°C. Islets were hand-picked under a stereomicroscope and a single-cell suspension prepared by treating the isolated islets with trypsin-EDTA (Life Technology, Grand Island, NY) (Brennand et al., 2007). β cells were cytofluorometrically sorted on cell size and autofluorescence (Dirice et al., 2013). Transcript quantification and data analysis were as above.

### In vitro suppression assays

TCRβ^+^CD4^+^CD25^−^ naïve T cells were sorted from spleen and labeled with 10 μmol/l CFSE (Molecular Probes) in RPMI 1640 at a concentration of 10^6^/ml at 37°C for 20 min; then were washed, resuspended in complete culture medium (RPMI1640, 10% fetal calf serum, 2 mmol/l L-glutamine, penicillin/streptomycin, and 2-mercaptoethanol), and cultured at 1 × 10^5^ cells/well in round-bottom, 96-well plates (Corning, Corning, NY). T cells were activated with anti-CD3/CD28 beads (at a ratio of 1:1 between cells and beads [Invitrogen]) and IL-2 (20 U/ml). Pancreatic myeloid cells from I-BET151-, or DMSO-treated mice were sorted into CD11b^+^CD11c^low/−^ or CD11b^low/−^ CD11c^+^ fractions, and were added to cultured T cells at a ratio of myeloid:T cells of 1:4. Similarly, CD4^+^CD25^+^ cells were sorted from I-BET151-, or DMSO-treated mice, and were added to cultured T cells at a ratio of 1:2. Proliferation was measured by flow-cytometric analysis of CFSE dilution, and division indices were calculated by computing the weighted fractions of all the divisions as per (D'Alise et al., 2008).

### Human monocyte-derived MFs

Blood from three healthy volunteers was used to isolate buffy coats by Ficoll–Paque density gradient centrifugation. Human monocytes were purified by positive selection of CD14-expressing cells (Miltenyi Biotec, UK). They were cultured in RPMI-1640 supplemented with 5% heat-inactivated fetal calf serum (Hyclone, ThermoScientific, UK), 2 mM Glutamine (Invitrogen, UK), 100 U/ml penicillin and 100 mg/ml streptomycin containing either 5 ng/ml GM-CSF (R&D Systems, UK) (condition 1) or 100 ng/ml M-CSF (R&D Systems, UK) (condition 2) and cultured for 5 days to generate MFs, respectively. After 5 days, MFs were treated with fresh medium containing either 0.1% DMSO or 1 μM I-BET151 for 30 min and then stimulated with 100 ng/ml LPS (L4391, Sigma, UK), or left unstimulated. Cells were harvested at 4 hr for extracting total RNA for transcriptional profiling.

Human biological samples were sourced ethically, and their research use was in accord with the terms of the informed consents.

### Immunostaining and microscopy

For visualizing β-cell proliferation, we injected EdU (1 mg) ip 24 hr before harvesting pancreata. Paraffin sections of pancreas 6 μm in thickness were prepared. Standard procedures were used for immunostaining (Fu et al., 2012b). Before the addition of primary mAbs, sections were blocked with 5% normal donkey serum (Jackson ImmunoResearch, West Grove, PA), then incubated overnight at 4°C with anti-insulin (Linco Research, St. Charles, MO), followed by FITC-AffiniPure Donkey Anti-Guinea Pig IgG (Jackson ImmunoResearch). EdU staining was performed as above. Nuclei were stained with DAPI (4′,6-diamidino-2-phenylindole dihydrochloride). Images were acquired on an Axiovert 200M confocal microscope (Zeiss, Peabody, MA) with a xenon arc lamp in a Lambda DG-4 wavelength switcher (Sutter Instrument), and were processed with Slidebook imaging software (Intelligent Imaging, Denver, CO).

### Statistical analysis

Tests used for statistical analyses are described in the various figure legends (GraphPad software v5.0; Prism, La Jolla, CA). p values of 0.05 or less were considered to be statistically significant.

## Additional files

Supplementary file 1. Genes over-represented in pancreatic CD45^+^ cells of NOD mice treated with I-BET151. **DOI:** http://dx.doi.org/10.7554/eLife.04631.011

Supplementary file 2. Genes under-represented in pancreatic CD45^+^ cells of NOD mice treated with I-BET151. **DOI:** http://dx.doi.org/10.7554/eLife.04631.012

Supplementary file 3. Expression of NF-κB target genes in pancreatic CD45^+^ cells of NOD mice treated with I-BET151. **DOI:** http://dx.doi.org/10.7554/eLife.04631.013

Supplementary file 4. Transcripts over-represented in β cells of NOD mice treated with I-BET151. **DOI:** http://dx.doi.org/10.7554/eLife.04631.014
